# Bacterial Membrane Vesicles: Physiological Roles, Infection Immunology, and Applications

**DOI:** 10.1002/advs.202301357

**Published:** 2023-06-25

**Authors:** Yixiao Gan, Gang Zhao, Zhicheng Wang, Xingcai Zhang, Mei X. Wu, Min Lu

**Affiliations:** ^1^ Department of Transfusion Medicine Huashan Hospital Fudan University Shanghai 200040 P. R. China; ^2^ Department of Orthopaedics Shanghai Key Laboratory for Prevention and Treatment of Bone and Joint Diseases Shanghai Institute of Traumatology and Orthopaedics Ruijin Hospital Shanghai Jiao Tong University School of Medicine Shanghai 200240 P. R. China; ^3^ John A. Paulson School of Engineering and Applied Sciences Harvard University Cambridge MA 02138 USA; ^4^ Wellman Center for Photomedicine Massachusetts General Hospital Department of Dermatology Harvard Medical School, 50 Blossom Street Boston MA 02114 USA

**Keywords:** antibiotic resistance, drug delivery, immune system, membrane vesicles, vaccine

## Abstract

Bacterial or fungal membrane vesicles, traditionally considered as microbial metabolic wastes, are secreted mainly from the outer membrane or cell membrane of microorganisms. However, recent studies have shown that these vesicles play essential roles in direct or indirect communications among microorganisms and between microorganisms and hosts. This review aims to provide an updated understanding of the physiological functions and emerging applications of bacterial membrane vesicles, with a focus on their biogenesis, mechanisms of adsorption and invasion into host cells, immune stimulatory effects, and roles in the much‐concerned problem of bacterial resistance. Additionally, the potential applications of these vesicles as biomarkers, vaccine candidates, and drug delivery platforms are discussed.

## Graphical abstract

As illustrated in the graphics, bacterial membrane vesicle (BMV) contributes to the adhesion and invasion of bacteria into the host cells, takes part in the interactions between bacteria and the innate immune system, also plays active roles in the development of bacterial resistance. Lastly, BMVs serve as valuable sources of biomarkers and novel nanoplatforms for vaccine delivery and drug therapy.

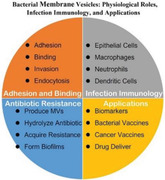

## 1 Introduction

Bacteria release various membrane‐bound materials, often termed membrane vesicles, microvesicles, or exosomes. According to the natures of membrane vesicles, bacterial vesicles secreted by Gram‐negative bacteria are commonly called outer‐membrane vesicles (OMVs), while secretory vesicles of Gram‐positive bacteria and fungi are uniformity called membrane vesicles (MVs).^[^ ^1^ ^]^ Although OMVs have similar contents to the outer‐membrane envelope of Gram‐negative bacteria, these vesicles are also heterogeneous depending on the living environment and contain specific bacteria‐associated adhesins, virulence factors, and/or RNA related to the gene expression required for adaptive responses to specific bacterial life activities or the exterior environment.^[^ ^1^ ^]^ For instance, *Moraxella catarrhalis* OMVs are enriched in adhesin, whereas *Helicobacter pylori* OMVs are enriched in UspA1 BabA, SabA, and VacA.^[^ ^2^ ^]^

Different from Gram‐negative bacteria, Gram‐positive bacteria consist of the cell wall and membrane yet lacking an outer membrane. The cell wall is composed of a block of peptidoglycan that can limit the release of vesicles for Gram‐positive bacteria.^[^ ^3^ ^]^ It was not evidenced until 1990 that MVs could be produced by Gram‐positive bacteria.^[^ ^4^ ^]^ Subsequently, transmission electron microscopy (TEM) and proteomic analysis have revealed direct evidence of vesicle formation from Gram‐positive bacteria.^[^ ^5^ ^]^ The MVs of Gram‐positive bacteria are rich in short‐chain saturated fatty acids, such as *Streptococcus pneumonia* and *Bacillus anthracis* that produce vesicles that are enriched in myristic and palmitic acids compared to bacterial cell membranes.^[^ ^6^, ^7^ ^]^ More importantly, *Staphylococcus aureus*‐derived MVs are particularly enriched in secretive or membrane‐associated virulence proteins including superantigens, hemolysins, Staphopain A, coagulation factors, IgG‐binding protein SbI, lipase, *β*‐lactamase, and N‐acetylmuramoyl‐l‐alanine amidase. These MV‐associated proteins play critical pathophysiological functions in communications in inter‐bacteria or between bacteria and host.^[^ ^5^ ^]^

The sizes of OMVs and MVs are 40–400 nm in diameter, but most vesicles from fungi are between 20 and 500 nm in diameter.^[^ ^8^ ^]^ For the latter, due to the thick cell wall, the research had not been deeply launched until the fungal vesicles were described and characterized in the pathogen *Cryptococcus neoformans*.^[^ ^9^ ^]^ Since then, a series of fungal secreted vesicles have been studied, for instance, *Histoplasma capsulatum* ^[^ ^10^ ^]^ and *Candida albicans*.^[^ ^11^ ^]^ The main sterol‐derivatives detected in fungi are ergosterol and lanosterol, whereas the most abundant neutral glycosphingolipid in fungi is glucosylceramide.^[^ ^9^, ^11^, ^12^, ^13^ ^]^

Over the years, the study of bacterial membrane vesicles (BMVs) has typically focused on their functions, particularly in association with their pathogenesis. Apart from proteins and lipids, small genetic molecules, such as mRNA, small RNA (miRNA and siRNA), and genomic DNA are also found to be encapsulated in vesicles and these vesicles take part in pathogenesis and in escaping from host immune cells through the silencing of specific gene expression and suppressing of the immune system.^[^ ^14^, ^15^, ^16^, ^17^ ^]^ Overall, the proteins, lipids, a series of virulence factors that vary with the source bacteria, and newly discovered RNA molecules packed in BMVs all have their specific functions, such as adhesion of cells, penetration of mucosal layer, expansion of infections, suppression of immunity, etc.^[^ ^18^ ^]^ It is worth noting that the same bacteria can produce different types of vesicles via distinct biogenetic pathways, which holds truth for both Gram‐positive and Gram‐negative bacteria, mycobacteria, and fungi.^[^ ^8^, ^19^ ^]^ In addition to their contribution to bacterial infections and pathogenesis, BMVs are increasingly attractive to be new delivery nanoplatforms for various vaccines and drugs, a hot area of research in the past decade.^[^ ^20^ ^]^

In the current review, we summarize the roles of various BMVs in infections, antibiotic resistance, and pathogenesis, and discuss recent advances with respect to their applications in a reversal of antibiotic resistance, vaccine designs, and drug delivery. The types of vesicles are diverse, and the research of these vesicles is in all dimensions because of their potential in many areas of medical applications. We believe that the understanding of the pathogenesis and clinical potentials of BMVs just begins and further exploration of this line of research should confer unique opportunities for more effective therapies of infectious diseases, cancers, and other disorders.

## 2 Biogenesis Pathways of Bacterial Membrane Vesicles

**Figure 1:**
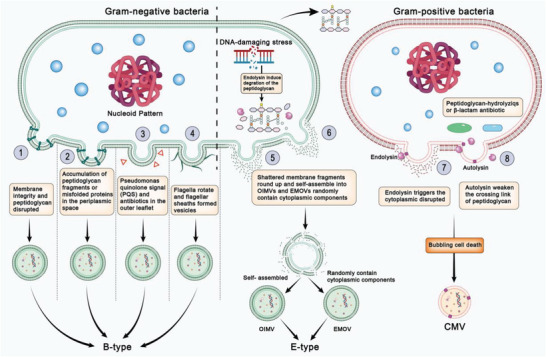
Schematic diagram of different biogenesis of bacterial membrane vesicles. 1) Crosslinking between peptidoglycan and the outer membrane integrity is disrupted due to various stressful responses of bacteria. 2) Accumulation of peptidoglycan fragments or misfolded proteins in the periplasmic space. 3) Quorum‐sensing molecule *Pseudomonas* quinolone signal (PQS) or antibiotics destabilize the biochemical structure. 4) Bacterial flagella surrounded by a sheath rotating along the membrane blebs. 5) Shattered membrane fragments round up and self‐assemble into OIMVs from explosive cell lysis triggered by genotoxic stress. 6) Cytoplasmic components randomly assembled into EOMVs. 7) Endolysins weaken peptidoglycan and trigger the production of explosive CMVs. 8) Autolysins weaken the crosslinking of the peptidoglycan and modulate CMV release through the cell wall.

BMVs can be produced by both Gram‐negative and Gram‐positive bacteria through different pathways.^[^ ^8^, ^19^, ^21^ ^]^ The two primary pathways are 1) that the blebbing of membrane materials of living bacterial cells gives rise to classic MVs (B‐type MVs) and 2) that endolysin‐triggered bacterial cells’ explosion degrades the bacterial peptidoglycan layer yielding explosive outer‐membrane vesicles (E‐type MVs) (**Figure** **1**).^[^ ^8^ ^]^ The different biogenetic modalities affect the contents of MVs as a consequence of a specific and complex physiological process.

### 2.1 BMV Formation through Membrane Blebbing

For Gram‐negative bacteria, classic OMVs are conventionally formed by the blebbing of the outer membrane and therefore do not have direct access to cytoplasmic contents as B‐type OMVs (Figure 1).^[^ ^19^ ^]^ The OMV release has been assumed to occur through three mechanisms. One of the most common mechanisms is the disruption of crosslinking between peptidoglycan and the outer membrane as a consequence of stressful responses to various disturbances such as imbalance of peptidoglycan biosynthesis, causing dissociation of the outer membrane from the peptidoglycan layer^[^ ^22^ ^]^ (Figure 1①). *Escherichia coli* mutants with crosslinking defects produce more OMVs than corresponding *E. coli* wild‐type strains. *E. coli nlpI* mutant with a subtle defect in crosslinking (a reduction of approximately 40% compared with wild‐type cells) was shown to hyper vesiculate without detectable cell lysis.^[^ ^23^, ^24^ ^]^ A recent study showed that phospholipid accumulation in the outer leaflet of the outer membrane of *Haemophilus influenzae* promoted the OMV release without compromising membrane integrity.^[^ ^25^ ^]^

The second mechanism for OMV formation through blebbing is the accumulation of peptidoglycan fragments or misfolded proteins in the periplasmic space (Figure 1②) as has been demonstrated in *E. coli* ^[^ ^26^ ^]^ *and Pseudomonas aeruginosa* strains.^[^ ^27^ ^]^ Moreover, integration of the quorum‐sensing molecule *Pseudomonas* quinolone signal (PQS) can destabilize biochemical structure that induces membrane curvature into the cell envelope of bacteria (Figure 1③).^[^ ^28^, ^29^ ^]^ The accumulation of PQS in the outer leaflet is thought to change the membrane curvature, introducing the initial driving force for OMV formation.^[^ ^30^ ^]^ Besides PQS, some antibiotics, such as gentamicin, and the cationic antimicrobial peptides polymyxin and colistin, can cause membrane perturbations, and induce OMV formation as well.^[^ ^31^ ^]^ This pathway will be described again later.

The third mechanism for OMV formation is unique to the bacterium assembling flagella. The flagella are surrounded by a sheath derived from the outer membrane. Membrane blebs are commonly found along the sheathed flagella and released when the flagella rotate (Figure 1④).^[^ ^32^ ^]^ Likewise, *Vibrio cholerae* was shown to use its sheathed flagella to release OMVs carrying lipopolysaccharide (LPS), which can trigger inflammation and an immune response in the host.^[^ ^32^ ^]^ The flagellar of *H. pylori* filaments could be disintegrated by acid treatment and the resultant flagellar sheaths formed vesicles, sometimes with characteristic structures.^[^ ^33^ ^]^ These observations suggest that flagellar‐mediated LPS release through OMVs may be common among bacteria with sheathed flagella.

### 2.2 BMV Formation through Explosive Cell Lysis and Bubbling Cell Death

Explosive cell lysis is triggered by genotoxic stress that activates the expression of prophage‐derived endolysins, which degrade the bacterial peptidoglycan layer both in Gram‐negative and Gram‐positive bacteria.^[^ ^34^, ^35^ ^]^ As a consequence, the cells round up and explode yielding shattered membrane fragments that round up and self‐assemble into E‐type MVs.^[^ ^34^, ^35^ ^]^ DNA‐damaging stress induces the expression of endolysin in *P. aeruginosa*, which is part of a pyocin biosynthesis gene cluster, and the endolysin further degrades the peptidoglycan layer.^[^ ^36^ ^]^ Usually, once the peptidoglycan is degraded, the cell rounds up and explodes, and then, the shattered membrane fragments round up and self‐assemble into outer‐inner membrane vesicles (OIMVs) (Figure 1⑤) or explosive outer‐membrane vesicles (EOMVs) (Figure 1⑥). In contrast to OMVs formed by blebbing, EOMVs randomly contain cytoplasmic components.^[^ ^37^, ^38^ ^]^

Similarly to Gram‐negative bacteria, Gram‐positive bacteria protrude cytoplasmic membrane (CM) materials through the holes in the peptidoglycan, which are then released as explosive cytoplasmic membrane vesicle (CMV).^[^ ^39^, ^40^ ^]^ The expression of an endolysin encoded by a defective prophage triggers the formation of CMVs in *Bacillus subtilis* (Figure 1⑦).^[^ ^35^ ^]^

Although the enzymatic activities of the endolysins weaken peptidoglycan in microbes, the consequences differ. Whereas *P. aeruginosa* cells undergo complete disintegration, the thick Gram‐positive cell wall of *B. subtilis* is not entirely hydrolyzed, although most cells die owing to a loss of membrane integrity caused by the endolysins and autolysins, evidenced by the formation of ghost cells and intracellular CMVs.^[^ ^8^, ^35^ ^]^ The phenomenon is also named “bubbling cell death”, which is frequently found in other Gram‐positive bacteria such as *Lactococcus lactis*,^[^ ^41^ ^]^ *Streptococcus suis*,^[^ ^42^ ^]^ and group A *Streptococcus*.^[^ ^43^ ^]^

Besides endolysin, autolysins are another factor induced under stress conditions by peptidoglycan‐hydrolyzing enzymes or *β*‐lactam antibiotics (Figure 1⑧). For example, CMV biogenesis in *S. aureus* is proposed to occur via a blebbing mechanism, which involves the CM disruptions by amphipathic, *α*‐helical, or phenol‐soluble modulins.^[^ ^44^, ^45^ ^]^ Subsequently, autolysins, which weaken the crosslinking of peptidoglycan, modulate the CMV release through the cell wall.^[^ ^45^, ^46^ ^]^

## 3 Adhesion, Invasion, and Immunization of Bacterial Membrane Vesicles

**Figure 2:**
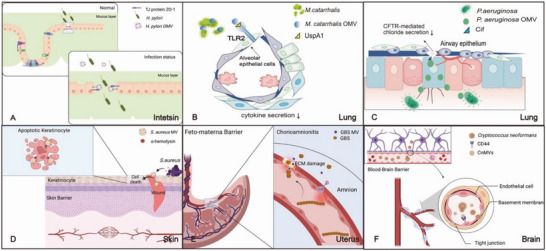
Schematic illustration of BMVs’ invasion in the mucosal barriers. A) *H. pylori* OMVs containing CagA can directly interact with the TJ protein ZO‐1 and open the cellular junction for the bacteria crossing the intestinal mucosal barrier. B) In lung, *M. catarrhalis* OMVs complete the adsorption process into alveolar epithelial cells by TLR2 on cell surface and the cytokine secretion decreased. C) *P. aeruginosa* OMVs delivering Cif that significantly help *P. aeruginosa* alleviate the mucociliary clearance and C if fuse with airway epithelial cells lipid raft domains in lung. D) *S. aureus* MVs’ immunostimulatory cargo like *α*‐hemolysin can induce keratinocyte cell death, exacerbate infected wounds, and disrupt the skin barrier. E) GBS MVs encapsulated with EMC can disrupt amniotic epithelium and reduce collagen synthesis in feto‐maternal interface damaging the barrier. F) *C. neoformans*‐derived microvesicles activate the surface film of HBMECs by interaction with receptor CD44 and break the blood‐brain barrier (BBB). A, D, E, F) created with BioRender.com.

MVs shed by Gram‐positive bacteria or OMVs released by Gram‐negative bacteria can support the survival of the bacteria and spread their infections by assisting their competition of nutrients with host cells, invasion of host tissues, and escape from immune surveillance. Studies have constantly shown that MVs and OMVs contribute to better adaption of the bacteria to the host's environment. By enriching virulence factors like LPS, lipids, outer membrane proteins, periplasmic proteins, enzymes, toxins, peptidoglycan, and sometimes, the bacterial DNA or RNA, and the ability to deliver them over long distances, the pathogens can invade host tissues and sufficiently compete for nutrients with host cells.^[^ ^8^, ^39^, ^47^, ^48^, ^49^ ^]^ Moreover, the expression of some key inflammatory factors can be either up or down regulated to allow them escaping the immune surveillance. Our emphasis on this section is the molecular mechanism underlying the bacterial invasion of mucosal barriers, a major process for bacterial infections (**Figure** **2**).

### 3.1 Adhesion and Initial Binding of Vesicles to Host Cells

The infection starts when bacteria stick to mucous epithelial cell membrane in the gut or the respiratory tract. MVs and OMVs mediate the adherence of host cells and act as virulence factor delivery vehicles for bacteria to continue invading at this point.^[^ ^50^ ^]^ As discussed below, their interaction can occur via adhesin‐receptor‐mediated attachment by virulence factors.^[^ ^51^ ^]^ In addition to intracellular signaling of the host cell, there are innate and adaptive immune responses in the host.^[^ ^52^, ^53^, ^54^ ^]^

As an illustrative example, *H. pylori*, a Gram‐negative pathogen, specifically colonizes the epithelial lining in the stomach of about 50% of the human population.^[^ ^55^ ^]^ Persistent infections with *H. pylori* are invariably associated with a mild chronic inflammation of the gastric mucosa. Major protein components of *H. pylori* vesicles have been confirmed including *α*‐subunit of urease, the cytotoxin vacuolating cytotoxin A (VacA), cytotoxin associated gene A (CagA), the blood group antigen‐binding adhesion (BabA), and sialic acid‐binding adhesin (SabA).^[^ ^2^ ^]^ VacA, CagA, and UreA are three major virulence factors of *H. pylori*.^[^ ^56^, ^57^, ^58^ ^]^ Among the virulence factors, several known and putative adhesins were detected in OMVs, for instance, HopZ and HorB proteins, BabA, BabB, SabA, SabB, *H. pylori* adhesin A (HpaA), and adherence‐associated lipoproteins (AlpA, AlpB).^[^ ^58^, ^59^, ^60^ ^]^ The finding of adhesins on OMV surfaces is in a good agreement with their roles in the binding and uptake of OMVs by gastric epithelial cells.^[^ ^51^ ^]^ One of the well‐studied mechanisms for *H. pylori* OMVs’ invasion is CagA that can alter the mucosal barrier by modulating the permeability of cellular junctional complexes. OMVs can directly interact with TJ protein ZO‐1 opening the cellular junction so that the bacteria can cross the intestinal mucosal barrier as illustrated in Figure 2A.^[^ ^49^, ^61^ ^]^ A new study shows that *H. pylori* OMV may contribute to the onset of Alzheimer's disease (AD) through complement component C3a receptor (C3aR) signaling.^[^ ^62^ ^]^ *H. pylori* OMVs could enter the brain via a transcellular pathway crossing the barriers or indirectly via the vagus nerve^[^ ^63^ ^]^ and increased the compactness and surface area of A*β* plaques in mice, which is an important pathological features of AD. It's because of the C3‐C3aR signaling pathway that *H. pylori* OMV can increase astrocyte‐microglia crosstalk and microglia activation triggering the neuronal dysfunction, A*β* deposition, and ultimately cognitive impairment.^[^ ^62^, ^64^ ^]^

Apart from brain, *M. catarrhalis*, a human respiratory pathogen, causes otitis media in children and exacerbates chronic obstructive pulmonary disease (COPD).^[^ ^65^, ^66^ ^]^ *M. catarrhalis* OMVs contain ubiquitous surface proteins (Usp) A1/A2 and Moraxella IgD‐binding protein (MID). The latter assists the interaction of bacteria with human B cells by clustering IgD B cell receptors into lipid raft motifs.^[^ ^53^ ^]^ Likewise, the interactions between epithelial cells and *M. catarrhalis* OMVs, lipid raft motifs are also observed in epithelial cells. In this process, TLR2 is compartmentalized into lipid rafts, in concert with the autotransporter proteins UspA1 and MID to facilitate adhesion to host cells. In combination with alveolar epithelial cells, MID is indispensable but not UspA1. The latter binds more to TLR2 and acts to inhibit cytokine secretion^[^ ^67^ ^]^ (Figure 2B).

*P. aeruginosa*, an opportunistic human pathogen, is frequently linked to nosocomial infections, particularly ventilator‐associated infections and pseudomonal pneumonia in immunocompromised patients with cystic fibrosis (CF). It is the primary reason for the death of patients with CF.^[^ ^68^, ^69^ ^]^ *P. aeruginosa* live anaerobically in the mucus layer of the CF lung and are rarely found in direct contact with epithelial cells. In the absence of direct cell‐to‐cell contact, *P. aeruginosa* OMVs have been proposed to be the actual contact. Four secreted factors alkaline phosphatase, *β*‐lactamase, hemolytic phospholipase C, and CFTR inhibitory factor (Cif) of *P. aeruginosa* have been reported to be packaged in OMVs.^[^ ^70^ ^]^ Cif is associated with reduced lung function in CF because Cif inhibits CFTR‐mediated chloride secretion in the airways thereby reducing mucociliary clearance^[^ ^71^ ^]^ (Figure 2C). Because cilium‐mediated physical removal of bacteria to prevent them from adherence to the epithelial cells is a key barrier in the respiratory tract, Cif embedded in OMV conquers this barrier and facilitates *P. aeruginosa* invasion via its long‐distance delivery by OMVs.^[^ ^72^ ^]^ Cif‐OMVs fuse with airway epithelial cell lipid raft domains and deliver Cif to the cytosol to accomplish the physiological functions above.^[^ ^73^ ^]^

Unlike Gram‐negative bacteria, most Gram‐positive bacteria have a thick peptidoglycan cell wall outside of the cell membrane, which prevents the production of extracellular vesicles and also hinders researchers' efforts to understand the mechanism. Only a few Gram‐positive bacteria including *Bacillus cereus*,^[^ ^74^ ^]^ *B. subtilis*,^[^ ^75^ ^]^ *Streptococcus mutans*,^[^ ^76^ ^]^ *S. aureus*,^[^ ^77^ ^]^ *B. anthracis*,^[^ ^6^ ^]^ and *Mycobacterium ulcerans* ^[^ ^78^ ^]^ meet the target to produce MVs.

The most common Gram‐positive bacteria studied so far are *S. aureus*, which are the major concerns for outbreaks occurring within hospital settings owing to the development of multi‐drug resistance.^[^ ^79^, ^80^ ^]^ The protein composition of MVs produced by S. aureus was characterized by Nano‐LC‐ESI‐MS/MS showing a diverse array of bacterial components, including cytosolic, surface, and membrane proteins, as well as surface adhesins, lipoproteins, toxins, and even nucleic acids.^[^ ^5^, ^81^ ^]^ A number of studies have shown that *S. aureus* MVs’ immunostimulatory cargo can significantly drive inflammatory responses during *S. aureus* infection, which can accelerate the disruption of the epithelial barrier and allow them to colonize in host tissues.^[^ ^1^, ^77^, ^82^, ^83^, ^84^, ^85^ ^]^ *S. aureus* MVs harbor *α*‐hemolysin, which induces keratinocyte cell death, resulting in disruption of the skin barrier, by which pathogen‐associated antigens and allergens can penetrate the skin and subsequently colonize to affect the host immune responses^[^ ^86^ ^]^ (Figure 2D).

*Streptococcus agalactiae*, another opportunistic Gram‐positive pathogen, is associated with premature rupture of amniotic membrane and preterm birth.^[^ ^87^ ^]^ Group B *Streptococcus* (GBS) produces MVs that contain a certain amount of extracellular matrix (ECM) degrading proteases, leading to the disruption of amniotic epithelium and reduction in collagen in vivo. The findings suggest that GBS MVs can independently orchestrate events at the feto‐maternal interface causing chorioamnionitis, membrane damage, and the disruption of feto‐maternal barrier^[^ ^50^ ^]^ (Figure 2E).

Most fungi also have thick walls composed of chitin, *β*‐glucan, and mannoprotein outside the cell membrane. The virulence factor plus membrane polysaccharides were transported through vesicles derived from *C. neoformans*.^[^ ^9^ ^]^ The most important virulence factor is glucuronoxylomannan (GXM).^[^ ^88^ ^]^ Similar to mammals, fungal extracellular vesicles, which are also eukaryotes, are rich in RNA content and take part in regulating persistent infections.^[^ ^48^ ^]^ In addition, *C. meningoencephalitis‐* and *C. neoformans*‐derived microvesicles (CnMVs) can bind to the human brain microvascular endothelial cells (HBMECs) and cross the Blood‐brain barrier (BBB) breaking the barriers to spread the infection^[^ ^89^ ^]^ (Figure 2F). Although we do not know which virulence factor is involved in this process, CnMVs can promote neo‐cryptococcal adhesion and cell swallow by activating the surface film of HBMECs via binding to the receptor CD44, which alters the distribution of proteins and lipid rafts in the cell membrane.^[^ ^90^ ^]^

### 3.2 Membrane Vesicles Enter the Host Cells via Distinct Endocytosis Pathways

**Figure 3:**
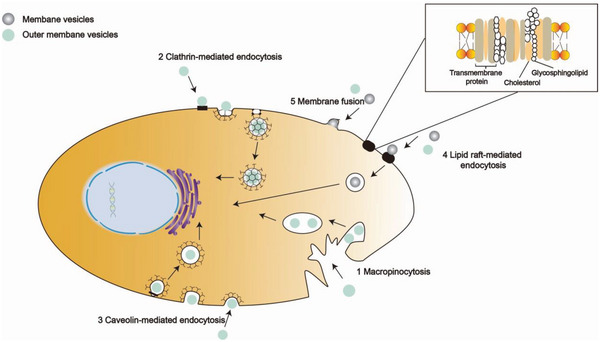
Schematic illustration of BMVs entering the host cells. 1) Macropinocytosis, which is driven by actin that form cup‐shaped membrane ruffles. When the ruffles fold back, they enclose the BMV. The closure followed with pinching off from the plasma membrane give rise to irregularly shaped vesicles and the vesicle will be released into the lysosome. 2) Clathrin‐mediated endocytosis. BMV recruitment concentrates cargo molecules to the coated region of the plasma membrane. The assembling coat promotes membrane bending, which transforms the flat plasma membrane into a “clathrin‐coated pit”, subsequently releasing the nascent cargo‐filled vesicle and allowing it to be trafficked within the cell. 3) Caveolin‐mediated endocytosis. Lipid raft domains can also be enriched in caveolin and the oligomerization of caveolin allows the formation of caveolae. Similar to clathrin‐mediated endocytosis, dynamin is also required for the scission and internalization of caveolae, but caveolin‐mediated endocytosis has higher efficiency than CME. 4) Lipid raft‐mediated endocytosis requires small GTPases in a protein receptor‐independent fashion to accept BMV into cells. 5) Membrane fusion. The lipids of BMV fuse with the cell phospholipid bilayer and the contents of the vesicle are then released into the cytoplasm.

After completion of the first step of the adhesion, the vesicles enter the host cell cytoplasm by different endocytosis pathways. Similar to what occurs in eukaryotic extracellular vesicles,^[^ ^91^ ^]^ there are five endocytosis pathways for BMVs to enter host cells: 1) macropinocytosis, 2) clathrin‐mediated endocytosis, 3) caveolin‐mediated endocytosis, and there are clathrin and caveolin‐independent mechanisms such as 4) lipid raft formation, and 5) membrane fusion. Owing to the heterogeneity of the vesicles themselves, even the same microorganism uses different ingestion pathways because vesicles vary in size and contents, we discuss how representative bacterial vesicles enter the host cells in different ways (**Figure** **3**).

#### 3.2.1 Macropinocytosis

Actin‐dependent macropinocytosis is characterized by the formation of large (over 200 nm in diameter) ruffled concavities from the cell membrane, which allow the sampling and internalization of extracellular materials^[^ ^92^, ^93^ ^]^(Figure 3①). Weiner et al. have studied invasion of *Shigella flexneri* OMVs into HeLa cells via macropinosomes by ion beam/scanning electron tomography (C‐FIB/SET).^[^ ^94^ ^]^ But the studies also tend to suggest that this cytosis effect is not the main one and there are more appropriate routes to intake the vesicles produced by most bacteria.^[^ ^93^ ^]^

#### 3.2.2 Clathrin‐Mediated Endocytosis

Clathrin‐mediated endocytosis (CME) occurs via the formation of clathrin‐coated pits up to 200 nm in diameter by utilizing toxin–receptor interactions to facilitate MVs’ cargo delivery^[^ ^95^, ^96^ ^]^ (Figure 3②). VacA in *H. pylori* is an important cytotoxic virulence factor and can be found in OMVs during infection. OMVs containing VacA enter host cells more efficiently than their VacA‐deficient counterparts, in the action with human gastric adenocarcinoma epithelial cells (AGS cells). A decrease of internalization efficiency can be demonstrated by using the inhibitor chloropropyl, which confirms that *H. pylori* vesicles can be internalized via CME.^[^ ^97^ ^]^ But the core component is clathrin, adaptor protein 2 (AP‐2), and dynamin in this process. AP‐2 is an adaptor protein required for the internalization of clathrin‐coated pits and dynamin is involved in the scission of clathrin‐coated pits from the plasma membrane and has also been suggested to be an important component in other endocytic pathways.^[^ ^98^ ^]^ Through colocalization analysis and small interfering RNA (siRNA) to specifically knock down individual target proteins‐AP‐2, the study found *H. pylori* to colocalize with dynamin II and clathrin in AGS cells. Combined with the previous inhibition experiments, it can be inferred that the *H. pylori* OMVs containing VacA have a similar co‐localization with *H. pylori* in this process.^[^ ^99^ ^]^ Besides, *Enterohemorrhagic E. coli* (EHEC) OMVs also have some functional links between clathrin and AP‐2 by LPS, which provide strong evidence for OMV to enter the host cell through the receptor‐mediated endocytosis.^[^ ^100^ ^]^

For Gram‐negative bacterium, LPS structure sometimes determines the preferred entry route of OMVs into host cells, not clathrin‐mediated endocytosis but the others.^[^ ^101^ ^]^ *Listeria monocytogenes* is a Gram‐positive bacterium responsible for listeriosis, and can cross the intestinal, blood‐brain, and placental barriers. *L. monocytogenes* is a facultative intracellular pathogen and can invade and replicate in epithelial cells and macrophages.^[^ ^102^ ^]^ L. monocytogenes invade various cell types, including nonphagocytic cells, by utilizing two internalins, internalin A (InlA) and internalin B (InlB). *L. monocytogenes* is able to interact with Met, a receptor on a nonphagocytic cell, and use soluble InlB to mediate clathrin‐dependent endocytosis of Met.^[^ ^103^ ^]^ Recent studies have determined that the classical virulence factors of L. monocytogenes, including InlA and InlB, are abundant in secreted MVs.^[^ ^104^ ^]^ It can be speculated, in reference to the toxicity experiments with mammalian cells, that MVs of *L. monocytogenes* enter the host cell via the CME pathway.

#### 3.2.3 Caveolin‐Mediated Endocytosis

Lipid raft domains can also be enhanced in caveolin and the oligomerization of caveolin allows the formation of caveolae (Figure 3③). Similar to clathrin‐mediated endocytosis, dynamin is also required for scission and internalization of caveolae but caveolin‐mediated endocytosis has a higher efficiency than CME.^[^ ^105^ ^]^ OMVs derived from non‐typeable *H. influenzae* were shown to enter and colocalize with caveolin 1 (Cav‐1) which is on behalf of a marker of caveolae.^[^ ^106^ ^]^ Cholera toxin (CTx) is a virulence factor of *V. cholerae* known to bind to the ganglioside GM1 presented in caveolin‐enriched lipid rafts on the host cell surface. During infection of intestinal epithelial cells, OMV‐associated CTx was found to rapidly target GM1 and facilitate the internalization of the OMVs.^[^ ^107^ ^]^

#### 3.2.4 Lipid Raft‐Mediated Endocytosis

Alternatively, lipid raft‐mediated endocytosis can be independent of caveolin and dynamin and instead requires small GTPases in a protein receptor‐independent fashion^[^ ^108^ ^]^ (Figure 3④). As mentioned above, LPS structure impacts the entry kinetics of bacterial OMVs into host cells. Research confirms that EHEC (with intact O antigen) sustained a higher entry rate over a longer period of time than non‐pathogenic *E. coli* K12 (O antigen deficient strain).^[^ ^101^, ^109^ ^]^ Although the lack of O antigens does not alter a maximal rate of entry (*r*_max_), the presence of the LPS O antigen sufficiently increases the entrance of OMVs into host cells because they can access raft‐mediated endocytosis more efficiently.^[^ ^101^ ^]^ In other words, when the intake time is long enough, the amount of the former and the latter into the host cell is similar, but within a certain period, the amount of EHEC OMVs entering into the host cell is greater. In addition, OMV‐associated proteases from *V. cholerae* were reportedly delivered into intestinal epithelial cells via this dynamin independent, lipid raft mediated endocytic route.^[^ ^110^ ^]^

Although nothing has been reported regarding the interaction of *S. aureus* MVs with host cells by specific cytotoxic molecules, a recent study found a similar mechanism, in which *S. aureus* MVs delivered MV components to host cells through the interactions with lipid raft machinery, suggesting a common entry mechanism for the MVs derived from Gram‐negative and Gram‐positive bacteria.^[^ ^111^ ^]^

The same mechanism is reflected in the internalization of fungal vesicles as the study observed host cell GM1, a raft marker, and proved a potential association between *C. albicans*‐derived MVs and lipid raft.^[^ ^11^ ^]^

#### 3.2.5 Membrane Fusion

The direct fusion of the vesicles with the host cell membrane has also been observed (Figure 3⑤). For example, *Legionella pneumophila*, *Aggregatibacter actinomycetemcomitans*, and *P. aeruginosa*, all of which transmit virulence factors into the host cells through membrane fusion.^[^ ^112^, ^113^ ^]^ The fusion of *S. aureus* MVs with eukaryotic cell membranes has also been clearly demonstrated with cholesterol‐dependent fusion of *S. aureus* MVs with the host cell plasma membrane instead of localizing to lipid raft microdomains.^[^ ^114^ ^]^

## 4 Bacterial Membrane Vesicles and Innate Immune System: Escape, Activate, and Counterattack

Bacterial colonization and every step along the infection are accompanied by a host immune response and bacteria interact with the human immune system through the cell‐to‐cell communication.^[^ ^115^, ^116^ ^]^ The BMVs that contain bacterial components play an important role in interacting with the innate immune system. During the life cycle of bacteria, in order to promote survival and diffusion of toxicity, the bacteria continuously produce vesicles in the hosts. After the host cells are infected, extracellular vesicles secreted from host cells to regulate immunity and inhibit infection. This bacteria‐host communication results in the alteration of different immune cell receptors and the secretion of cytokines listed in **Table** **1**.

**Table 1:** Cytokine changes in different natural immune cells

Species	Cell	Receptor	Inflammatory Factor
*E. coil*	Epithelial cells	TLR4	MyD88, NF‐*κ*B, IL‐1*β*↑^[^ ^120^, ^121^ ^]^
*P. aeruginosa*	Epithelial cells	TLR4/MD‐2	IL‐6, IL‐8, TNF‐*α*↑^[^ ^120^, ^122^ ^]^
		NOD1	IL‐8↑^[^ ^123^ ^]^
*H. pylori*	Epithelial cells	NOD1	IL‐8↑^[^ ^123^ ^]^
*N. gonorrhoeae*			
*V. cholerae*			
*S. aureus*	Epithelial cells	TLR2^a)^ & NOD2	IL‐8, IL‐1*β*, CCL2↑^[^ ^84^ ^]^
*P. aeruginosa*	Macrophages	NLRP3	IL‐1*β* & IL‐18↑^[^ ^54^, ^83^, ^130^, ^131^, ^132^, ^133^ ^]^
*B*. *pertussis*			
*E*. *coli*			
*P. gingivalis*			
*Mycobacterium*	Macrophages	TLR2	IL‐1*β* & TNF↑^[^ ^134^, ^135^ ^]^
*L. monocytogenes*	Macrophages	RIG‐I	IFN‐1↑^[^ ^147^ ^]^
*C. neoformans*	Macrophages		TNF‐*α*↑^[^ ^142^ ^]^
*A. fumigatus*			
*H. pylori*	Macrophages	TLR4	IL‐10 ↓^[^ ^143^, ^144^ ^]^
*P. gingivalis*			
*N. meningitidis*	Neutrophils		TNF & IL‐1*β*↑^[^ ^85^, ^150^ ^]^
*S. aureus*			
*A. baumannii*	Neutrophils	TLR2&TLR4	IL‐6↑^[^ ^151^ ^]^
*P. aeruginosa*			
*Salmonella* spp	Dendritic cells	CD86 & MHCII	TNF & IL‐12↑^[^ ^160^ ^]^
*Streptococcus*	Dendritic cells		TNF‐*α* & IL‐10 ↑^[^ ^163^ ^]^
*C. albicans*	Dendritic cells		IL‐12, IL‐10, TGF‐*β*, TNF‐*α* ↑^[^ ^11^ ^]^
*M. sympodialis*	Dendritic cells	ICAM‐1	IL‐4 & TNF‐*α* ↑^[^ ^166^ ^]^

^a)^ MVs containing DNA and RNA are detected by TLR7, 8, and 9.^[^ ^16^ ^]^

### 4.1 BMVs’ Interactions with Epithelial Cells

**Figure 4:**
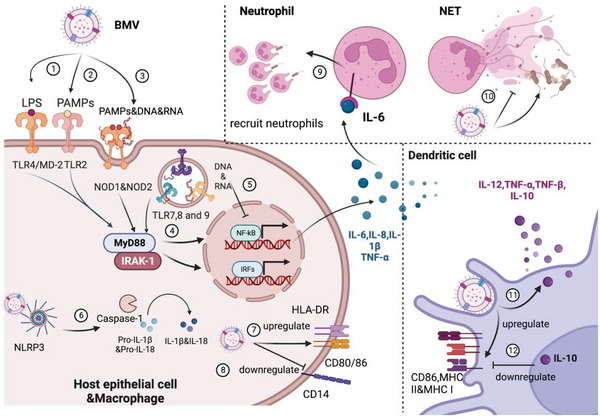
Schematic illustration of BMV interacts with different innate immune cells including epithelial cell, macrophage, neutrophil, and dendritic cell. In host epithelial cell and macrophage, BMV and its cargo (MAMPs, LPS, DNA and RNA) are recognized by TLRs and NLRs, resulting in the secretion of proinflammatory factors (IL‐6, IL‐8, IL‐1*β* and TNF‐*α*) and the up/down‐regulation in surface receptors. In neutrophil, IL‐6 secreted by macrophage combined with receptors to recruit neutrophils; Meanwhile, BMV induce NET formation to kill pathogens or degrade the structure of NETs. In dendritic cell, BMV is internalized into DCs, resulting in the secretion of IL‐12, TNF‐*α*&*β* and IL‐10; CD86 and MHC molecules are upregulated by BMV but downregulated by anti‐inflammatory factor ‐IL‐10. Created with BioRender.com

As described in the first part of this article, OMVs and MVs produced by pathogens can invade epithelial cells to break the epithelial barrier where pathogen recognition receptors on epithelial cells are the innate immune system's first responders to pathogenic microbes. BMVs contain numerous microorganism‐associated molecular patterns (MAMPs), DNA, RNA, lipoproteins, LPS, and peptidoglycan.^[^ ^117^ ^]^ The innate immune system defends against pathogens by sensing MAMPs with pattern recognition receptors (PRRs), such as the toll‐like receptors (TLRs), a class of PRRs that are most widely studied (**Figure** 4 ①–②).^[^ ^118^ ^]^ TLRs are classified into cell membrane‐associated and intracellular TLRs. The cell membrane‐associated TLRs respond to components of the microbial membrane such as proteins, lipids, and lipoproteins, while intracellular TLRs recognize bacterial and viral nucleic acids.^[^ ^119^ ^]^ NOD‐like receptors (NLRs) are also a family of intracellular innate PRRs that can detect BMVs contents like MAMPs (**Figure** **4** ③ ).^[^ ^117^ ^]^

*E. coli* OMVs contain LPS that can trigger TLR4‐dependent CXCL8 production in human epithelial cells and *P. aeruginosa* OMVs are shown to engage human epithelial cells in a TLR4‐dependent manner, resulting in the increased expression of TLR4 in a positive‐feedback mechanism to drive further inflammation, while also increasing host expression of MyD88, NF‐*κ*B, and IL‐1*β* (Table 1). LPS is a common component of Gram‐negative bacterial OMVs, its lipid A endotoxin is a MAMP that is detected by the TLR4/MD‐2 (myeloid differentiation factor 2) complex on host membranes through MD‐2, and the lipid A component of LPS and cardiolipins together bind TLR4 on host cells.^[^ ^120^ ^]^ The interactions of TLR4 on host cells lead to the phosphorylation and nuclear translocation of the transcription factor NF‐*κ*B and the production and release of pro‐inflammatory cytokines IL‐6, IL‐8, TNF‐*α*, and the like (Figure 4 ④–⑤).^[^ ^121^ ^]^

In addition to TLR4, experiments with MEFs from nucleotide‐binding oligomerization domain‐containing protein 1 (NOD1)‐deficient mice proved that OMVs from *Neisseria gonorrhoeae*, *P. aeruginosa*, *H. pylori*, and *V. cholerae* activate pro‐inflammatory signaling via NOD1 and were taken up by epithelial cells.^[^ ^122^ ^]^ OMVs can enter non‐phagocytic cells such as epithelial cells and their peptidoglycan fraction can interact with NOD1. This interaction promotes autophagy and the release of inflammatory cytokines like IL‐8^[^ ^123^ ^]^ (Table 1).

Of particular interest is TLR2, which plays an important role in controlling innate immune responses by communications with numerous microbial structures.^[^ ^124^ ^]^ A recent study suggested that *S. aureus* MVs, which contained abundant proteins, DNA, RNA and peptidoglycan, could effectively activate nucleotide‐binding oligomerization domain‐containing protein 2 (NOD2) via TLR2 in a dose‐dependent manner.^[^ ^84^ ^]^ TLR4 is traditionally considered a predominant receptor for Gram‐negative bacterial LPS. It is noteworthy that *S. aureus* peptidoglycan can also activate TLR4, albeit to a lesser extent, besides TLR2 and NOD2.^[^ ^125^ ^]^ Moreover, *S. aureus* MVs can mediate a pro‐inflammatory response in epithelial cells because MVs can stimulate A549 human lung epithelial cells to produce IL‐8, IL‐1*β*, and CC‐motif ligand 2^[^ ^84^ ^]^ (Table 1).

Different from peptidoglycan and LPS, nucleic acid components are able to activate intracellular TLRs, such as TLR9 that recognizes unmethylated CpG motifs which are prevalent in bacteria but not vertebrate genomic DNAs.^[^ ^16^ ^]^ Similarly, bacterial RNA and DNA in BMVs are distinguished by intracellular TLR7, 8, and 9, respectively. In addition to TLRs, *S. aureus* MVs containing DNA and RNA are also detected by NOD2 innate immune receptors resulting in NF‐*κ*B activation.^[^ ^84^ ^]^ Moreover, a role of RNA in escaping the host immune system seems convincing as has been reflected in the studies of BMVs and host epithelial cells.

*P. aeruginosa*‐derived methionine tRNA can be transferred into human epithelial airway cells via OMVs, resulting in a decrease in IL‐8 secretion and aggregation of neutrophils and macrophages onto the site of infection so that bacterial clearance can be slowed down. As we mentioned above, the virulence factor Cif dampened the airway innate immune response by promoting lysosomal degradation of CFTR reducing chloride secretion, so did sRNAs from *P. aeruginosa* derived‐OMVs. OMVs transferred sRNAs to host cells, which diminished OMV‐stimulated IL‐8/KC secretion by human airway epithelial cells thus reducing the clearance ability of the innate immune system.^[^ ^126^ ^]^ More recently, a role for small non‐coding RNAs (sncRNA) in *H. pylori* pathogenesis and evasion of the gastric epithelial cells has been identified, whereby sncRNAs can also attenuate IL‐8 secretion.^[^ ^127^ ^]^

In addition to suppression of pro‐inflammatory cytokines and aiding to escape the host immune system, BMV‐mediated PRR signaling can stimulate the production of antimicrobial peptides by host epithelial cells and facilitate the bacterial clearance. OMVs from *N. gonorrhoeae*, *P. aeruginosa*, and *H. pylori* induced epithelial cell expression of the human‐*β*‐defensins HBD2 (also known as DEFB4A) and HBD3 (also known as DEFB103A), both of which have direct antimicrobial effects.^[^ ^128^ ^]^

### 4.2 BMVs’ Interactions with Macrophages

After crossing the epithelial barrier, BMVs can modulate innate immune cells. One of the important cell types is macrophages which survey host tissues and rapidly detect and respond to invading pathogens. The cells phagocytose the bacteria and BMVs and also secrete various products provoking downstream immune responses. On the other hand, macrophages can serve as host cells for certain pathogenic microorganisms. BMVs of these intracellular microorganisms interact with various components within the host cells in a complicated manner, which is ill understood today. The pro‐inflammatory or anti‐inflammatory effect seems to be relevant to species and stages of infection and sometimes bacteria‐infected macrophages also release extracellular vesicles that contain pathogen‐derived macromolecules and affect other immune cells.^[^ ^129^ ^]^ Our focus is on *Mycobacterium* and *L. monocytogenes* BMVs that typically invade macrophages and stimulate other immune cells as well.

It is clear that an entrance of BMVs into macrophages can drive pro‐inflammatory cytokine production. The pro‐inflammatory responses involve NOD1, NOD2, and several other nucleotide oligomerization domain‐like receptors (NLRs). These NLRs function as microbial sensors within the innate immune system such as the NLR family known as NLR‐pyrin domain containing 3 (NLRP3). The activation of NLRP3 by BMVs leads to the formation of an intracellular complex termed the inflammasome, a protein complex responsible for the maturation of caspase‐1 and the subsequent secretion of mature IL‐1 family cytokines including IL‐1*β* and IL‐18.^[^ ^83^ ^]^ *P. aeruginosa*,^[^ ^54^ ^]^ *B*. *pertussis*,^[^ ^130^ ^]^ *E*. coli,^[^ ^131^ ^]^ *P. gingivalis* OMVs^[^ ^132^ ^]^ and *S. aureus*,^[^ ^83^ ^]^ *S. pneumonia* MVs^[^ ^133^ ^]^ can activate NLRP3 inflammasome in murine bone marrow‐derived macrophages (BMDMs) or human macrophages (THP‐1 cells and human monocyte‐derived macrophages) and promote releases of the mature cytokines IL‐1*β* and IL‐18 (Figure 4 ⑥). Functionally, *Mycobacterium* MVs have been shown to induce the production of IL‐1*β* and TNF by macrophages through TLR2, and in turn *Mycobacterium avium*‐infected macrophages release extracellular vesicles that can stimulate a pro‐inflammatory response in non‐infected or naïve macrophages, which is also dependent on TLR2.^[^ ^134^ ^]^ *Mycobacterium tuberculosis* (Mtb)‐infected macrophages secrete a number of extracellular vesicles containing mycobacterial proteins, including LpqH, which is present on host‐derived extracellular vesicles (hEVs). LpqH is the TLR2 ligand and the primary driver of this inflammatory response.^[^ ^134^, ^135^ ^]^ Moreover, *Salmonella enterica* OMVs can activate macrophages and dendritic cells via TLR4.^[^ ^52^ ^]^ Initial work has indicated that THP‐1 cells infected with *S. enterica* serovar Typhimurium release hEVs that stimulate TNF production in a TLR4‐dependent manner.^[^ ^136^ ^]^ More recently, this effect appears macrophage specific, as EVs derived from infected macrophages induce TNF secretion in naïve macrophages and dendritic cells, whereas EVs from infected dendritic cells do not elicit such a response in either cell type. Perhaps, it is the combination of LPS in hEVs with TLR4 of other macrophages that dominate this process.^[^ ^52^, ^137^ ^]^ MVs from pathogenic fungi also increase cytokine production in macrophages as *C. neoformans* and *A. fumigatus* are internalized by murine macrophages, producing TNF‐*α*.^[^ ^138^, ^139^ ^]^

Second, BMVs activate macrophages in association with adaptive immune responses increasing bacterial killing. *N. meningitidis* OMVs upregulate the expression of HLA‐DR, the costimulatory molecules CD80, CD86, and intercellular adhesion molecule 1 (ICAM‐1) by macrophages (Figure 4 ⑦).^[^ ^140^ ^]^ Moreover, mycobacterial‐infected macrophages release hEVs containing mycobacterial RNA that activates the host RIG‐I/MAVS/TBK1/IRF3 RNA sensing pathway and promotes the maturation of Mtb‐containing phagosomes, resulting in increased bacterial killing.^[^ ^141^ ^]^ Similarly, *A. flavus* augmented cytokine production in bone marrow macrophages and facilitated the fungicidal activity of the macrophages.^[^ ^142^ ^]^

Last, BMVs can modulate the immune response mediated by macrophages through a switch of their pro‐inflammatory phenotypes to anti‐inflammatory phenotypes with which bacteria can survive longer within the host and also permit secondary bacterial infections. For example, *H. pylori* OMVs induce IL‐10 production in human peripheral blood mononuclear cells^[^ ^143^ ^]^ while *P. gingivalis* OMVs facilitate a loss of CD14 expression on macrophages (Figure 4 ⑧),^[^ ^144^ ^]^ rendering these cells unresponsive to TLR4 signaling and effectively avoiding hyperinflammatory immune responses. For the pathogen with chronic infection, the mechanism is more complex as *L. pneumophila* OMVs are initially potent pro‐inflammatory stimulators of macrophages via TLR2, IRAK‐1, and NF‐*κ*B, followed by facilitating *L. pneumophila* replication by miR‐146a‐dependent IRAK‐1 suppression.^[^ ^145^ ^]^ *Mycobacterial*‐infected macrophages release two groups of cellular vesicles which could suppress the immune response and are originated from the secretion of intracellular *Mycobacterium*.^[^ ^141^ ^]^ Indeed, hEVs from Mtb‐infected cells partially suppress the ability of recipient macrophages to respond to IFN‐*γ* and also inhibit downstream CD4^+^ T cell activation through the lipoarabinomannan (LAM) which is also present in Mtb BMVs.^[^ ^129^, ^146^ ^]^ Meanwhile, *L. monocytogenes* MVs can activate type‐1 IFN production through the RIG‐I cytosolic RNA‐sensing pathway via ril32, a highly conserved *L. monocytogenes* RNA that is packaged into MVs thus promoting bacterial survival in macrophages.^[^ ^147^ ^]^

### 4.3 BMVs’ Interactions with Neutrophils

The interactions between BMVs and neutrophils can be categorized into two groups: immunomodulatory effects and neutrophil extracellular trap (NET)‐related effects.^[^ ^148^, ^149^ ^]^ Foremost, direct stimulation of human neutrophils with OMVs from *N. meningitidis* and *S. aureus* MVs results in induction of TNF and IL‐1*β* and upregulation of CXCL8, CCL3, and CCL4 expression.^[^ ^85^, ^150^ ^]^ Indirectly, neutrophils can migrate to the site of infection in response to chemo‐attractants and chemokines released by epithelial cells or macrophages. For example, *A. baumannii* ^[^ ^151^ ^]^ and *L. plantarum* ^[^ ^152^ ^]^ OMVs can activate TLR2 and TLR4 on the surface of macrophages in the lungs via the common adaptor protein MyD88 to promote the release of IL‐6. IL‐6 is a proinflammatory cytokine secreted by macrophages and can recruit neutrophils which play central roles in the clearance of infectious organisms during acute infection (Figure 4 ⑨).^[^ ^153^ ^]^

One of the hallmarks of neutrophil death is the production of neutrophil extracellular traps (NETs), which contain DNA, antimicrobial peptides, and histones together forming extracellular fibers to trap and kill extracellular pathogens.^[^ ^154^, ^155^ ^]^ BMVs from *N. meningitidis*, *P. aeruginosa*, and *Streptococcus pneumoniae* can induce NET formation to kill these pathogens (Figure 4 ⑩).^[^ ^156^ ^]^ However, some of them like *S. pneumoniae* can evade NET entrapment by packaging the extracellular DNase TatD into MVs to degrade the structure of neutrophil NETs.^[^ ^157^ ^]^

### 4.4 BMV Interactions with Dendritic Cells

Dendritic cells (DCs) are central players in the immune response in bridging the innate and adaptive immune systems.^[^ ^158^, ^159^ ^]^ Bacteria and bacteria‐derived BMVs have been shown to be internalized into DCs up‐regulating CD86 and MHCII molecules on DCs and producing TNF and IL‐12.^[^ ^160^ ^]^ In *B. fragilis* OMVs have been shown able to stimulate DCs to produce pro‐inflammatory factors and cytokine.^[^ ^161^ ^]^ The OMVs require IBD‐associated genes, *ATG16L1*, which is a CD (spell out CD here)‐risk gene specifically expressed in CD11c^+^ DCs, to protect against experimental colitis. Under similar conditions, *ATG16L1*‐deficient mice failed to induce regulatory T cells (Tregs) and suppress mucosal inflammation and OMVs could not sufficiently mitigate the colitis.^[^ ^161^ ^]^ In addition, *M. tuberculosis* MVs also substantially increase the expressions of MHC‐I, MHC‐II, and CD86 on DCs, further demonstrating that PAMPs in Mtb MVs induce DC maturation.^[^ ^162^ ^]^

It is important to note, however, that the effects of DCs and BMV are heterogeneous, meaning that they may have different effects with different microorganisms, due to the type of bacteria or TLR they receive. For example, recent research has revealed that *streptococcal* MVs can be internalized by DCs, resulting in increased secretion of TNF‐*α* and IL‐10 instead of IL‐12 (Figure 4 ⑪).^[^ ^163^ ^]^ IL‐10 possesses a broad anti‐inflammatory activity and it inhibits antigen presentation, decreases MHCII expression, and hinders DC differentiation from precursor monocytes (Figure 4 ⑫).^[^ ^164^, ^165^ ^]^ The seeming contradiction also manifests itself in MVs released by fungi. *C. albicans* MVs can significantly stimulate the production of IL‐12, IL‐10, TNF‐*α*, and TGF‐*β* in DCs^[^ ^11^ ^]^ and the MVs from *M. sympodialis* can augment the ICAM‐1 expression and promote IL‐4 and TNF‐*α* responses in DCs.^[^ ^166^ ^]^

## 5 Bacterial Membrane Vesicles and Antibiotic Resistance

**Figure 5:**
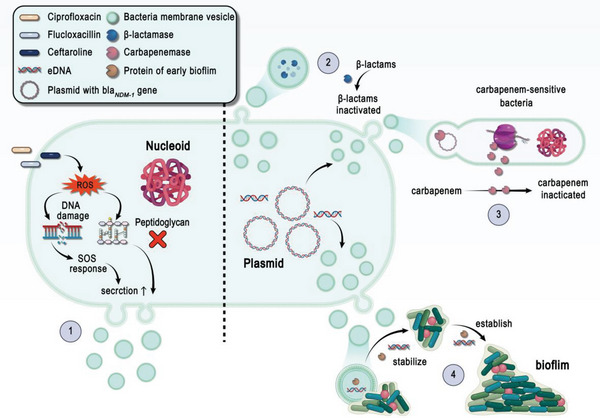
A schematic of the mechanisms involved in antibiotic resistance and bacterial vesicles. 1) On the left of the dotted line, ciprofloxacin triggers the SOS response to weaken the Gram‐positive bacterial cell wall, concomitant with strong induction of vesicle formation by flucloxacillin or ceftaroline. 2) On the right of the dotted line, BMVs can carry antibiotics contained with *β*‐lactamase and glycopeptides functional groups and further neutralize or hydrolyze antibiotics. 3) BMVs can mediate horizontal gene transfer (HGT) to help bacteria acquire antibiotic resistance. and 4) BMVs can help bacteria stabilize and establish biofilms and further induce the broad‐spectrum resistance to antibiotics.

Overprescription of antibiotics and the continuous evolution of bacteria have led to the emergence of multidrug‐resistant strains of clinical important pathogens. As we have repeatedly emphasized, BMVs play unique roles in bacterial life cycle and infection, including influence on bacterial resistance through different mechanisms in order to prolong survival. Currently, there are five known mechanisms of antibiotic resistance:, i.e., 1) the active generation of one or more inactivated enzymes; 2) the mutations of antibiotics’ targets, like penicillin‐binding protein (PBP); 3) a decrease of bacterial membrane permeability; 4) the overexpression of efflux system; and 5) the change of metabolic pathway or the establishment of metabolic bypass. The mechanism that vesicles contributing to antibiotic resistance appears closely related to the above mechanisms as summarized in **Figure** **5**.

### 5.1 Antibiotics Activate Bacteria to Produce Membrane Vesicles

Bacteria trigger a series of stress responses to external conditions by which they adapt to various environments. Antibiotics can be considered one of the stresses provoking vesicle production (Figure 5①). For example, *P. aeruginosa* exposed to gentamicin produced three times as many OMVs as it did in the absence of gentamicin.^[^ ^167^ ^]^ Quinolones and *β*‐lactam could increase BMV production both in *P. aeruginosa* ^[^ ^29^ ^]^ and *S. aureus*.^[^ ^168^ ^]^ OMV production in *P. aeruginosa* increases when treated with ciprofloxacin and flucloxacillin, so does *S. aureus* in response to ceftaroline. Ciprofloxacin leads to the activation of SOS response, a conserved regulatory network that is activated in response to DNA damage. Meanwhile, *β*‐lactam antibiotics can weaken the Gram‐positive bacterial cell wall so that the membrane materials can protrude into the extracellular space and are released as BMVs.^[^ ^18^, ^47^ ^]^ In addition to increased yields, there is also evidence showing that antibiotic usage alters the quality of BMVs. For instance, not only can ceftazidime and imipenem induce a higher number of BMVs produced by *A. baumannii* but also these BMVs carry more LPS and induce higher expression levels of iNOS, IL‐1*β*, and IL‐6 in macrophages.^[^ ^169^ ^]^ The same results were attained with *Burkholderia cepacia*.^[^ ^170^ ^]^

### 5.2 BMVs Can Neutralize or Hydrolyze Antibiotics

In *E. coli* or *P. syringae*, the addition of OMVs results in immediate resistance to the antimicrobial peptides polymyxin B, colistin, and melittin since proteases and peptidases encapsulated within OMVs can sequester or degrade these peptides directly.^[^ ^18^, ^171^ ^]^ BMVs can also carry various enzymes that mediate antibiotic resistance (Figure 5②). *M. catarrhalis* OMVs^[^ ^172^ ^]^ carry active *β*‐lactamase, and *Enterococcus faecium* MVs^[^ ^173^ ^]^ contain glycopeptides that hydrolyze bacterial peptidoglycan. Notably, BMVs mentioned above usually protect other bacterial species that are sensitive to a particular antibiotic during co‐survival or incubation. Recently, OMVs from amoxicillin‐resistant *M. catarrhalis* were found to carry active *β*‐lactamase and protect amoxicillin‐sensitive *M. catarrhalis* from antibiotic‐induced killing.^[^ ^172^ ^]^ Moreover, OMVs from amoxicillin‐resistant *M. catarrhalis* have also been shown to strengthen the amoxicillin resistance of non‐typeable *H. influenzae* and *S. pneumoniae*.^[^ ^172^, ^173^, ^174^ ^]^

### 5.3 Bacteria Acquire Antibiotic Resistance through BMVs

BMVs can mediate horizontal gene transfer (HGT) to help bacteria acquire antibiotic resistance and virulence genes (Figure 5③), which is well described in a recent review of spreading resistance genes both between the same species and different species.^[^ ^175^ ^]^ Carbapenem‐resistant clinical strains of *A. baumannii* can transfer the bla_OXA‐24_ gene and metallo‐*β*‐lactamase‐1 (bla_NDM‐1_) gene to carbapenem‐sensitive *A. baumannii* or *E. coli* JM109 via OMVs.^[^ ^175^ ^]^ Some mutants can enhance the release of membrane vesicles that function to transfer resistance genes to surrounding bacteria such as three variants of *Salmonella Typhi* that produce OMVs to protect *S. Typhi* WT from polymyxin B in a concentration‐depending manner.^[^ ^176^ ^]^

### 5.4 Involvement of BMVs in Biofilm Formation and Antibiotic Resistance

BMVs can assist bacteria to form biofilms that protect microbes within the biofilms and shield them from antimicrobial attacks (Figure 5④). A number of studies have demonstrated that BMVs are essential in the maintenance of the structural integrity of biofilms. For instance, *H. pylori* OMVs carried a unique 22 kDa protein of early biofilms but disappeared during biofilm maturation, suggesting a role for this OMV protein in the initial stages of biofilm formation.^[^ ^177^ ^]^

Extracellular genomic DNA (eDNA) was also found to associate with the biofilm formation. *Francisella* spp.,^[^ ^178^ ^]^ *A. baumannii*, *P. aeruginosa*,^[^ ^29^ ^]^ and *S. aureus* ^[^ ^179^ ^]^ all produce BMVs with eDNA within, which is essential for initiation and stabilization of biofilms.^[^ ^18^ ^]^ It has also been found in fungi that a blockade on the release of MVs from *C. albicans* weakens the synthesis of biofilm matrix, rendering *C. albicans* highly sensitive to several antifungal drugs and reaffirming that MVs contribute to antibiotic resistance by supporting the formation of biofilms.^[^ ^180^ ^]^

## 6 Applications of Bacterial Membrane Vesicles

**Figure 6:**
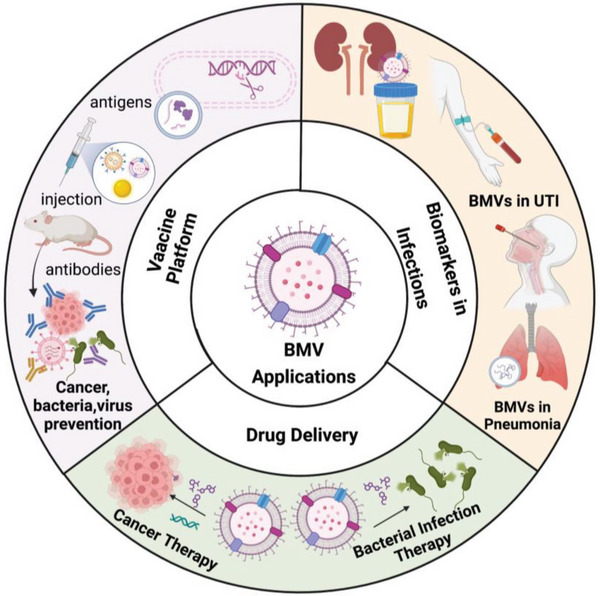
Potentials of biomedical applications of bacterial membrane vesicles in biomarkers, vaccine platform, and drug delivery. Created with BioRender.com.

As with live bacteria, BMVs are filled with a rich array of antigens, PAMPs, adhesins, proteins, DNA, RNA, and more.^[^ ^8^ ^]^ These components empower BMVs to exhibit remarkable biological capabilities. Especially, BMVs have been increasingly recognized for their potentials as infectious biomarkers, bacterial vaccines, adjuvants for virus and cancer vaccines, cancer immunotherapy agents, and drug delivery platform as summarized in **Figure** **6**.

### 6.1 BMVs and the Derivative as Biomarker Sources

Either membrane vesicles produced by bacteria or exosomes released by host cells as a consequence of bacterial infections have great potentials to be diagnostic biomarkers because they carry not only microbes‐specific but also host‐responsive molecular biomarkers and are not degraded easily in body fluids. At present, diagnosis of microbial infections still relies on bacterial cultures,^[^ ^181^ ^]^ which takes 2–3 d, and is well beyond the golden window of antibiotic treatment. If specific markers like the nucleic acids can be detected in the early stage of infection, it can greatly improve the efficiency of diagnosis and treatment and effectively prevent sepsis, a disease with a high death rate.

In this regard, clinical isolates from survivors of endovascular ST45/USA600 *Staphylococcal* infection produce significantly more MV than isolates from decedents, probably because low MV production is associated with reduced immune cell infiltration.^[^ ^182^ ^]^ The predictive value of vesicle production in the clinical mortality of MRSA is extended to *E. coli‐*mediated urinary tract infections (UTI). The bacterial and urothelial cell interactions lead to increased expression of CD9 and Akt (protein kinase B) in exosomes of host cells, suggesting that CD9 and Akt in urinary exosomes could be useful biomarkers for diagnosis of UTI and asymptomatic bacteriuria.^[^ ^183^ ^]^

Over the past decade, there has been increasing evidence that small non‐coding RNAs released by MVs can serve as diagnostic and prognostic markers for many diseases such as cancer, metabolic abnormalities, and cardiovascular disease.^[^ ^184^, ^185^ ^]^ When combined with observations that BMVs also carry RNA, RNA detection in bacterial vesicles or exosomes in body fluids should be well integrated into clinical microbiology lab workflows. In lung infections caused by Gram‐negative bacteria such as *Klebsiella pneumoniae*, the expression of miRNA‐223/142 in MV purified in alveolar lavage fluid was enhanced nearly 30‐fold.^[^ ^186^ ^]^ Compared with the detection of non‐EV‐containing miR‐223/142, MV‐containing miR‐223/142 potentially reflects the specific organs in which macrophages are being activated. These observations unravel a potential by which circulating MV‐miR‐223/142 can be used to predict lung inflammation and its dynamics postbacterial infections.^[^ ^186^ ^]^

Mi‐RNA can also play a key role in sepsis diagnosis. For example, miR‐223 could potentially help to distinguish non‐septic patients from septic ones^[^ ^187^ ^]^ and miR‐21 has been shown to be an essential part of the protective effect of remote ischemic preconditioning in sepsis.^[^ ^188^ ^]^ In addition, detecting miRNAs in TB patients and animals infected with *S. aureus* is available.^[^ ^189^ ^]^

### 6.2 BMV Is an Attractive Platform for Vaccine Development

BMVs naturally contain a range of highly immunostimulatory ligands and are strong drivers of the innate immune response. Because of their intact membrane structure, BMVs can protect the internal cargo from degradation by nucleases and proteases, apart from cost‐effectiveness, bioengineer ability, and stability even in prolonged cold temperatures, as demonstrated in the previous study.^[^ ^190^ ^]^ Intensive research over the last decade has suggested an immense potential and powerful platform of BMVs for vaccine delivery both in the prevention of infections and cancer therapy.

#### 6.2.1 Vaccines for Microbial Infections

##### Native BMV Vaccines Derived Directly from Bacteria

Vesicles directly isolated and purified from bacterial cultures can be used as vaccines, many of which have demonstrated immunogenicity. Bordetella pertussis MV vaccines showed that the MV vaccine raised antibody levels in mice at levels comparable to the current approved whole‐cell B. pertussis vaccine.^[^ ^130^ ^]^ Moreover, vaccination with *A. baumannii* OMVs increased the survival of mice in a sepsis model and significantly enhanced bacterial susceptibility to antibiotics in multiple murine models of infections.^[^ ^191^ ^]^ Besides *A. baumannii*, *S. aureus* often presents multidrug resistance, which is a serious problem around the world. Vaccination of mice with vesicles derived directly from *S. aureus* elicits a strong humoral immune response.^[^ ^84^ ^]^ The Meningitis type B (MenB)‐based vaccine is currently in clinical studies and on the market, which was prepared by using dissolved organic carbon detergent to release the vesicles.^[^ ^149^ ^]^ These called wild‐type MV vaccines have proved effective in places like Cuba,^[^ ^192^ ^]^ Brazil,^[^ ^193^ ^]^ and more recently in New Zealand.^[^ ^194^ ^]^ Some concerns about these natural vesicles derived directly from bacteria are associated with a potential loss of important antigenic proteins and/or impurity during the preparation process. So, bioengineered modification of BMVs may make vaccines more effective and safer.

##### Bioengineered and Modified BMV Vaccines

To address the antigenic incompleteness or LPS toxicity mentioned above, BMVs can be genetically engineered to increase the amounts of immune reactive determinants while minimizing toxins and deleterious compounds. For example, *S. aureus* MVs were modified by toxin gene mutation to express detoxified cytolysins like Hla and LukE to enhance the immunogenic potency.^[^ ^44^ ^]^ When the mice were immunized with *S. aureus* JE2*∆agr∆spa* eng‐EVs, JE2∆*agr∆spa* mutant Evs, and BSA, only the sera from mice immunized with *S. aureus* eng‐Evs effectively neutralized Hla, LukED, and HlgAB, and showed a significant protection against both *S. aureus* LAC strain and NRS685 strain infections in a lethal murine sepsis model.^[^ ^44^ ^]^ The study clearly demonstrates that recombinant proteins packaged within *S. aureus* EV are immunogenic.^[^ ^44^ ^]^

As we mentioned above, *B. pertussis* ^[^ ^130^ ^]^ and *A. baumannii* ^[^ ^195^ ^]^ OMVs containing a PagL‐deacylated modified LPS showed protection with lower reactogenicity than the corresponding B. pertussis and MenB vaccines. Multivalent porA‐based Men‐B OMV vaccine inserted with multiple porA genes has been developed based on genetically engineered strains exhibiting four times more efficiency in the induction of bactericidal antibodies than conventional vaccines.^[^ ^196^ ^]^ What is more, the second generation of MenB OMV vaccine is deficient in capsular polysaccharides while making penta‐acylated LpxL1 LPS that has a much lower endotoxic activity than the capsular polysaccharides. The modified Men‐B OMV vaccine strongly reduced IL‐6 production in human monocytes.^[^ ^196^, ^197^ ^]^

Qi et al. use gold nanoparticles (AuNPs) coated with *E. coli* OMVs to dramatically increase the stability of OMVs in vivo resulting in higher immunogenicity than un‐modified counterparts.^[^ ^198^ ^]^ Biomimetic nanoparticles like AuNPs or CuNPs provide stability and stimulate immune responses more efficiently than original BMVs.

##### Recombinant BMV Vaccines Assembled from Heterogeneous Vesicles

BMVs can also be engineered for vaccine development via incorporating heterologous antigens. The antigens or immunogens that can be genetically engineered into the MVs of other bacteria or the exosomes of different cells. *E. coli* OMVs have been frequently used as carriers for expressing heterologous polysaccharides. Based on ClyA fusion protein, Omp22 antigens from *A. baumannii* were fused into *E. coli* DH5*α*‐derived OMVs giving rise to high protection in a murine sepsis model.^[^ ^199^ ^]^ Likewise, OprI, *P. aeruginosa* A104R antigen was fused into *E. coli*‐derived OMVs as well.^[^ ^200^ ^]^

Apart from direct expression of heterologous antigens, exploitation of the lipoprotein transport machinery could be used to design recombinant vaccines as five *S. aureus* lipoproteins can accumulate in OMVs of *E. coli* by modifying genetically engineered transcription. This pentavalent OMV‐based *S. aureus* vaccine can induce antigen‐specific antibodies and protect mice from *S. aureus* Newman strain infection.^[^ ^125^ ^]^ In addition, engineered *S. enterica* and *E. coli* OMVs can also express the capsular and proteins of *S. pneumoniae* and induce protective antibodies to prevent pneumococcal colonization infection.^[^ ^201^, ^202^ ^]^

Even after modifications, the problems that cause systemic inflammatory responses and low OMV production remain, which have been illustrated well by the generation of bacterial protoplast‐derived nanosized vesicles (PDNVs) that are deprived of outer‐membrane components. PDNVs have built‐in adjuvanticity and can increase DC maturation and B and T cell responses. When PDNVs from *S. aureus* and *E. coli* were injected subcutaneously into mice, it showed a significant size effect, with PDNV less than 200 nm inducing a significant improvement in B‐cell antibody response, demonstrating a great potential for designing effective antibacterial vaccines in part by adjusting the size.^[^ ^203^ ^]^

**Figure 7:**
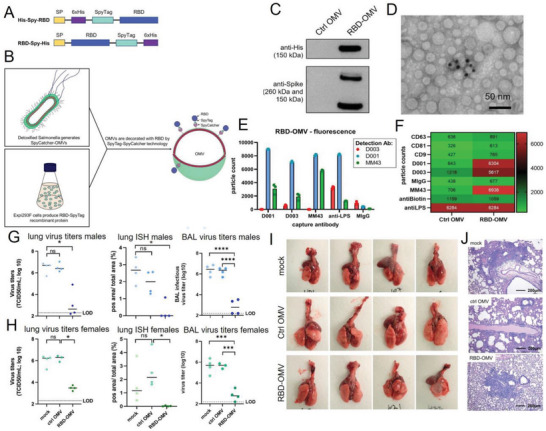
A BMV‐based intranasal vaccine elicits protective immunity against wild‐type and Delta variants of SARS CoV‐2. A) Design schematic of RBD recombinant antigens fused to N‐ and C‐terminal SpyTag. B) Schematic representation of the production of RBD‐OMVs. C) Western blot of Control‐ or RBD‐OMVs probed with anti‐His and ‐Spike antibodies. D) Immunogold TEM graphs with anti‐Spike‐MM43 and streptavidin‐gold. E) Labeling with fluorescently labeled antibodies D001, D003, and MM43 shows localization of CoV‐2‐Spike epitopes on RBD‐OMVs. F) Heatmap of SP‐IRIS data comparing RBD‐OMVs from (D) and Ctrl‐OMVs. G) Determination of virus titers and ISH in lung, and virus titer in BAL fluid among males. H) Measurements of virus titers and ISH in lung, and virus titer in BAL fluid among females. I) Gross examination of lungs from hamsters immunized with different formulations including mock RBD‐OMV vaccination (male group). J) Representative H&E staining of hamster lung sections from each experimental group. Reproduced with permission.^[^ ^204^ ^]^ Copyright 2022, Wiley.

The “plug‐and‐play” approach allows for the decoration of the same OMVs with a variety of antigens such as virus antigens. The approach was recently used to engineer SARS‐CoV‐2 vaccine candidates by decoration of Spike (S) receptor binding domains (RBD) on BMVs.^[^ ^204^ ^]^ The SpyTag‐RBD fusion protein was produced in mammalian cells and effectively coupled to *Salmonella typhimurium* OMVs by SpyTag/SpyCatcher system (**Figure** **7**A–F).^[^ ^205^, ^206^ ^]^ By vaccinating hamsters intranasally, SARS‐CoV‐2 virus titers and RNA scope ISH in the lung homogenate and BAL fluid were significantly reduced in the male (Figure 7G) or female (Figure 7H) hamster’ models. Furthermore, gross examination (Figure 7I) and H&E staining (Figure 7J) suggested that the lungs immunized with RBD‐OMV had fewer focal patches of inflammation and hemorrhagic areas after wild‐type and *Delta* SARS‐CoV‐2 challenges.^[^ ^204^ ^]^

#### 6.2.2 Vaccines for Cancer Therapy

Cancer vaccines can be divided into two types according to the clinical applications, preventive and therapeutic. Preventive vaccines are a novel immunotherapy of various cancers. The interaction between BMVs and the adaptive immune system starts with BMV crossing the mucous and draining via the lymphatic fluid to lymph nodes where they stimulate DC maturation and antigen presentation. This process is followed by the secretions of the anti‐tumor cytokine IFN‐*γ*, the productions of antigen‐specific CD4^+^, CD8^+^ T cells, and specific B cells.^[^ ^207^, ^208^, ^209^ ^]^ Cancer cells are specifically killed by the adaptive immune system stimulated by BMV vaccines. The applications about BMV tumor vaccines are demonstrated through several examples.

##### Tumor‐Specific Epitopes Engineered in BMV Vaccines

Overall, a protective immune response has been described with BMV immunotherapy alone, with a long‐term memory effect.^[^ ^210^ ^]^ The main mechanism of action appears to preferably accumulate BMVs within tumor tissue wherein they induce the production of the anti‐tumor cytokines CXCL10 and interferon IFN‐*γ*. Moreover, the genetically modified *E. coli* OMV, whose gene encoding lipid A acyltransferase (*msbB*) had been inactivated, was injected intravenously into mice with CT26 murine colon adenocarcinoma, significantly eliminating the tumor. When CT26 cells were inoculated into the mice again, the tumor failed to grow as a result of immune memory responses.^[^ ^211^ ^]^ Different tumor cell types and vesicles of *Lactobacillus* were also validated for their immunotherapeutic efficacy.^[^ ^212^ ^]^

Sometimes, OMV alone injection can cause a severe systemic inflammatory response, which requires certain material modifications, such as biomineralization. *E. coli* OMVs, after encapsulated by biocompatible calcium phosphate (CaP) and pH‐sensitive nanoshell, can be accumulated within the tumor for a long time, greatly improving the acidity of the tumor microenvironment. When combined with a photosensitizer agent, the OMVs can facilitate photothermal‐induced immunogenic cell death (ICD) in CT26 solid tumor murine model and 4T1 tumor murine model.^[^ ^213^ ^]^

**Figure 8:**
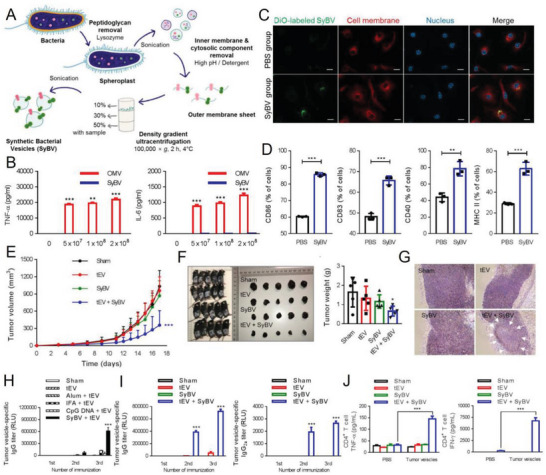
Synthetic bacterial vesicles combined with tumor extracellular vesicles as cancer immunotherapy. A) Schematic diagram of the isolation of *E. coli‐*derived SyBV. B) Levels of pro‐inflammatory cytokine TNF‐*α* (left) and IL‐6 (right) productions from RAW 264.7 cells treated with various doses of OMV or SyBV. C) Representative images of uptake of DiO‐labeled *E. coli* SyBV (green) by BMDCs at 6 h. D) Percentages of CD86^+^, CD83^+^, CD40^+^, and MHC II^+^ BMDCs treated with PBS and *E. coli* SyBV acquired from the flow cytometry results. E) Mice were s.c. immunized with tEV and/or SyBV for five times at 3‐day intervals following B16F10 inoculation. The tumor growth was monitored every 1 or 2 days. F) Pictures of mice and dissected tumors and tumor weight. G) Representative melanoma histology images on day 17 after immunization. Arrows indicate necrotic areas. H) Comparisons of the adjuvant activities of SyBV to that of other traditional adjuvants in terms of induction of human tEV‐specific IgG. I) Mice were i.p. injected with human tEV and/or SyBV for three times at weekly intervals, and then the human tEV‐specific IgG and IgG2a titers were measured in the blood. J) The levels of human tEV‐specific CD4^+^ T cell‐derived TNF‐*α* and IFN‐*γ* after CD4^+^ T cells were isolated from immunized spleens. Reproduced with permission.^[^ ^215^ ^]^ Copyright 2021, Wiley.

Besides direct applications as vaccines, BMVs can carry tumor antigens. Tumors, especially those with a large number of genetic mutations, carry “neo‐epitopes” that can become the targets of both CD4^+^ and CD8^+^ T cells. BMVs can be fused with tumor extracellular vesicles that are released either in situ or via circulation to obtain antigens.^[^ ^214^ ^]^ When combined with tumor extracellular vesicles, BMVs should be detoxified first through a process called synthetic bacterial vesicles (SyBV) or tumor cell vesicles (tEV) (**Figure** **8**A).^[^ ^215^ ^]^ The detoxified‐*E. coli* SyBV did not induce any toxicity to RAW 264.7 even at a much higher dose (Figure 8B,C). A combined immunization of the two types of vesicles significantly increased the infiltration of immune lymphocytes in tumor tissues, especially CD8^+^ T cells and NK cells, and inhibited tumor growth in melanoma (Figure 8D–G). Interestingly, human tEV‐specific immunity is stimulated by SyBV to a greater extent than other commercial adjuvants such as Alum, IFA, and CpG DNA, whereas human tEV alone did not affect the immunity (Figure 8H–J).^[^ ^215^ ^]^ OMV‐based in situ vaccine using the surface adsorption capacity of natural OMVs in combination with photothermal therapy (PTT) was able to capture the released tumor antigens effectively, followed by delivering the captured antigen to DCs and stimulating DCs to mature. These antigen‐specific T cells can suppress residual subcutaneous CT26‐luc tumor cells. Situ vaccine of 1‐MT@OMV‐Mal after PTT exhibited systemic effect to inhibit distant tumor growth through activating the systemic immune response against tumors.^[^ ^214^ ^]^

Genetic engineering and “plug‐in and Display” technologies to display the target antigens are needed for rapid expression of tumor antigens in different patient populations for precision tumor therapy. ClyA membrane melt antigen can be displayed on the surface of OMV and induce specific anti‐tumor immunity mediated by CD8^+^ T cells.^[^ ^216^ ^]^ Meanwhile, it can also successfully induce long‐term immune memory in mice, which has been verified in mouse melanoma model.^[^ ^208^, ^217^ ^]^

Other potential anti‐tumor targets include BMV vaccines that induce self‐production of antibodies to attack tumor cells, epidermal growth factor (EGFR) and the basic fibroblast growth factor (BFGF).^[^ ^218^ ^]^ Genetic modification technology reveals successful introduction of the full‐length mouse BFGF molecule onto *E. coil* OMVs and their breaking B cell tolerance in the body. BFGF‐modified OMVs induce high levels of anti‐BFGF autoantibodies suppressing TC‐1 and B16F10 tumor cell growth.^[^ ^219^ ^]^ On the other hand, a variant of EGFR‐EGFRvIII‐decorated *E. coil* OMVs was capable of inducing a potent anti‐EGFRvIII antibody response which strongly reduced the growth of B16F10 tumor cells expressing human EGFRvIII. Moreover, the Th1 profile induced by the OMVs favored the migration of IFN‐*γ*‐producing CD4^+^ and CD8^+^ T cells to the tumor site, essential to the protective activity of the vaccine.^[^ ^210^ ^]^

##### Small RNA‐Associated BMV Vaccine for Tumor Therapy

Therapeutic RNA vaccines have been recently developed rapidly attracting significant attention as an emerging option for tumor treatment. BMV mRNA vaccines have been validated in animal tumor models. “Plug‐in and Display” technology was also used to fuse mRNA and *Listeria* lysin O (LLO) to the C‐terminal of ClyA protein.^[^ ^208^, ^220^ ^]^ *E. coil* OMV + mRNA^ADPGK^ significantly inhibited melanoma progression and resulted in 37.5% complete regression in a colon cancer model. Moreover, the combined group induces a long‐term immune memory and protects the mice from tumor challenges after 60 d.^[^ ^221^ ^]^

##### BMV Vaccine for Programmed Cell Death

All of the BMV vaccines mentioned above induce strong IFN‐*γ* production, which, while strongly associated with cancer immunity, also upregulates immunosuppressive factors in the tumor microenvironment, especially programmed death 1 ligand 1(PD‐L1). PD‐L1 impedes T‐cell function and limits immunotherapy effectiveness. Li et al. have engineered OMV‐PD1 to bind PD‐L1 on the surface of tumor cells and promote its internalization and reduction, thus reversing the inhibition of T‐cell proliferation in a way similar to anti‐PD‐L1 antibody therapy but in a much more cost‐effective manner.^[^ ^209^ ^]^ OMV‐PD‐L1 has the immune stimulation ability of natural OMVs and can inhibit tumor growth in CT26 colorectal cancer mice and B16 melanoma mice with an immune memory that protects against tumor recurrence.^[^ ^209^ ^]^

### 6.3 BMVs as Nanoplatforms for Drug Delivery

Over the past five decades, there was an increasing trend of developing resistance to antibiotics or cancer drugs. Therefore, there is an urgent need to improve the biopharmaceutical properties of existing compounds and search for new and more effective antibiotics or pharmaceuticals. BMVs encapsulated with various drugs could be part of the solutions owing to their more targeted properties than free drugs and effective focused treatment at the site of infections and tumor. Importantly, the new delivery platform can significantly reduce the therapeutic dose of drugs and side effects and facilitate the effective concentration accumulated within the target due to the properties of membrane fusion.

In certain cases, it may be possible to directly harness naturally derived BMVs for a therapeutic outcome as BMVs from some species that package therapeutically relevant cargoes, such as antimicrobial peptides against competing species, and lend themselves particularly well for this purpose. For example, pseudomonas vesicles contain the autolysin murein hydrolase, which is capable of lysing other Gram‐negative and Gram‐positive bacteria. In this regard, MVs themselves can be described in a sense as a new class of “antibiotics” for specific bacterial infections.^[^ ^222^ ^]^

Current delivery of antibiotics to suppress infections has been focused primarily on biomimetic polymeric nanoparticles, using certain nanocarriers with some specific modifications of the synthesis of antibiotics aimed at specifically killing of targeted bacteria. If BMVs are purified directly from bacterial culture, a drug‐containing vesicles can be obtained by electroporation or by adding the corresponding antibiotic during culture such as the incorporation of gentamicin during growth of the *P. aeruginosa* 01 (PA01), which promoted gentamicin‐loaded BMVs and deliver the drug to the target bacterium *Burkholderia cepacia*.^[^ ^222^ ^]^

As for therapeutic BMVs for cancers, OMVs are engineered with siRNA targeting kinesin spindle protein (KSP), which can target and kill cancer cells in a cell‐specific manner.^[^ ^223^ ^]^ Moreover, a mutant *E. coli* strain was engineered to generate OMVs with a human epidermal growth factor receptor 2 (HER2)‐specific affibody in the membrane as a targeting ligand. Systemic injection of the siRNA‐packaged OMVs caused targeted gene silencing and induced highly significant tumor growth regression in an animal model with nonspecific side effects.^[^ ^224^ ^]^ While OMVs could activate the host immune response for cancer immunotherapy, the loaded drug within polymeric micelles would exert both chemotherapeutic and immunomodulatory roles to sensitize cancer cells to cytotoxic T lymphocytes (CTLs) and to kill cancer cells directly. Furthermore, by selectively delivering tegafur, a prodrug of fluorouracil (5‐FU), to solid tumor site through tumor‐targeted *Salmonella* OMV‐coated polymeric micelles, tegafur was found to be readily transformed into 5‐FU to exert its chemotherapeutic role to kill cancer cells and the embedded drug could also actively accumulate at the melanoma site.^[^ ^225^ ^]^ In addition, monoclonal antibodies and other anticancer drugs such as Doxorubicin have also been reported to be delivered by *S. enterica* serovar Typhimurium OMVs to treat tumors.^[^ ^211^, ^226^ ^]^ Bioengineered OMVs show great promise as cell‐specific drug‐delivery vehicles for treating various cancers.

## 7 Summary

Although BMVs have been studied for almost 4 decades, only in recent years have we witnessed the great advances in the understanding of the mechanisms by which BMVs contribute to bacterial‐triggered inflammation and pathology and of their ability to modulate the immune system by suppressing inflammation thus facilitating the survival of their parent bacterium in the host. These vesicles are derived from bacteria or fungi and contain a series of contents that are highly similar to bacterial cytoplasm, with slight differences in the amounts and species, and they regulate many physiological and pathological processes. Compared with bacteria themselves, BMVs act more like an “advance guard” and transport among bacteria and between bacteria and host cells to deliver virulence and adhesion factors, which are pivotal to bacterial adhesion and invasion of host cells. At the same time, these BMVs inevitably pass information to the host immune system and provoke immune responses including the innate immune system, particularly macrophages. The inhibition of immune system as the advantage for bacteria is also a function performed by many types of bacteria‐derived vesicles. The process not only depends on bacteria‐derived vesicles but also promotes host cells to produce extracellular vesicles containing contents similar to bacteria‐derived vesicles consequently playing a synergistic role in suppressing immune system.

In addition to the mRNA molecules and vesicles that inhibit the immune system, vaccine modification, and drug delivery have been a hot topic of research lately and this review also summarizes some examples of direct modifications of bacterial vesicles or biomimetic vesicles as therapeutic platforms both for infections and cancers. Although extensive studies have been carried out successfully in preclinical studies and provided enormous information about their clinical potentials, BMVs or MVs have not yet entered in clinical practices sufficiently. This is in part because the consistency of vesicle preparation from batch to batch still requires extensive investigation to completely avoid sepsis, which can be caused by high concentrations of LPS and other side effects owing to the impurity of the vesicles. Moreover, large‐scale BMV production associated with drug delivery or vaccine application requires mature manufacturing, storage, and transportation technologies. To date, there is no consistent industry standard for the manufacture of BMV‐related products. More efforts are needed to reach the level of current mature industrial preparations. In the future, specific roles for RNA molecules encapsulated in the BMVs and clinical trials for BMV vaccines and drug delivery remains to exploit. The stability and specific targeting of BMVs have great potential in future drug delivery platforms for anti‐cancer drugs and even other diseases, as well as applications of siRNA. We look forward to future research that can address these questions and increase our understanding of these complex and interesting bacterial derivatives so as to better fight bacterial infections and cancers in humans.

## Conflict of Interest

The authors declare no conflict of interest.

## Author Contributions

Y.G.: conceptualization, review, providing graphic materials, writing—original draft. Z.W.: conceptualization, funding acquisition, writing—review & editing. G.Z.: writing—original draft. X.Z.: writing—review & editing. M.X.W.: supervision, writing—review & editing. M.L.: conceptualization, supervision, funding acquisition, writing—review & editing.
